# Mutation Burden of Rare Variants in Schizophrenia Candidate Genes

**DOI:** 10.1371/journal.pone.0128988

**Published:** 2015-06-03

**Authors:** Simon L. Girard, Patrick A. Dion, Cynthia V. Bourassa, Steve Geoffroy, Pamela Lachance-Touchette, Amina Barhdadi, Mathieu Langlois, Ridha Joober, Marie-Odile Krebs, Marie-Pierre Dubé, Guy A. Rouleau

**Affiliations:** 1 McGill University and Genome Quebec Innovation Center, McGill University, Montréal, Canada; 2 Montreal Neurological Institute and Hospital, Department of Neurology and Neurosurgery, McGill University, Montréal, Canada; 3 Centre de Pharmacogénomique Beaulieu-Saucier, Institut de Cardiologie de Montréal, Montreal, Canada; 4 Université de Montréal, Faculty of Medicine, Montréal, Canada; 5 Département de pathologie et biologie cellulaire, Université de Montréal, Montreal, Canada; 6 Université Paris Descartes, Faculté de Médecine Paris Descartes, Paris, France; INSERM U894, Laboratoire de Physiopathologie des Maladies Psychiatriques, Paris, France; 7 Service Hospitalo Universitaire, Centre Hospitalier Sainte-Anne, 7 rue Cabanis, 75014 Paris, France; 8 Douglas Mental Health University Institute, Department of Psychiatry, McGill University, Montréal, Canada; Odense University hospital, DENMARK

## Abstract

**Background:**

Schizophrenia (SCZ) is a very heterogeneous disease that affects approximately 1% of the general population. Recently, the genetic complexity thought to underlie this condition was further supported by three independent studies that identified an increased number of damaging *de novo* mutations DNM in different SCZ probands. While these three reports support the implication of DNM in the pathogenesis of SCZ, the absence of overlap in the genes identified suggests that the number of genes involved in SCZ is likely to be very large; a notion that has been supported by the moderate success of Genome-Wide Association Studies (GWAS).

**Methods:**

To further examine the genetic heterogeneity of this disease, we resequenced 62 genes that were found to have a DNM in SCZ patients, and 40 genes that encode for proteins known to interact with the products of the genes with DNM, in a cohort of 235 SCZ cases and 233 controls.

**Results:**

We found an enrichment of private nonsense mutations amongst schizophrenia patients. Using a kernel association method, we were able to assess for association for different sets. Although our power of detection was limited, we observed an increased mutation burden in the genes that have DNM.

## Introduction

Schizophrenia (SCZ) is a highly prevalent neurodevelopmental disorder (1.1% of U.S. adult according to NIMH), that severely affects social and vocational development, and that has a strong negative stigmatization. Late adolescence and early adulthood is the peak period for the onset of SCZ (typically ~15–25 yrs)[1]. Moreover, according to the World Health Organisation (WHO), nearly half of the patients with SCZ are not receiving appropriate healthcare. The contribution of genetics to SCZ has been widely examined and a recent meta-analysis of 12 twin studies established heritability to be ~81%[2]. A simplistic view of the genetic architecture of SCZ suggests it involves different common alleles with low penetrance, intermediate frequency alleles with variable penetrance, and/or rare but highly penetrant alleles. More recently, a new hypothesis has emerged for SCZ: the implication of de novo mutations (DNM, i.e. mutations arising sporadically either in the gamete cells of the parents or at the early stage of embryo development) as a source of rare penetrant variants. The development of high-throughput sequencing technologies facilitated the systematic genome-wide testing of this DNM based hypothesis. First, using sanger sequencing of SCZ trios (a proband plus his/her mother and father) our group found that patients with SCZ and autism spectrum disorder have a higher than expected exonic DNM rate, as well as a high nonsense/missense ratio very similar to pathogenic Mendelian mutations[3]. Using an exome sequencing strategy we replicated these finding in trios of cases with sporadic SCZ[4]. An independent study by Xu *et al*. produced similar results thus validating our findings[5]. The same group later reported a new DNM study for which they exome sequenced many additional trios with a proband affected with SCZ[6]. They observed an excess of nonsynonymous DNM as well as a higher prevalence of gene-disruptive DNM. Another team also identified several DNM in SCZ patients and mapped several of those genes to a gene network pointing to the fetal prefrontal cortical neurogenesis[7]. The importance of DNM in neurodevelopmental diseases has been converging in other psychiatric disorders (e.g. autism[8–10] and intellectual disability[11]). However, the individual relevance of genes found to harbour a DNM is still unknown. Thus, we decided to follow-up on these genes to establish if they have a role in the biological mechanism of SCZ.

## Methods

### Cohorts

This study was reviewed and accepted by the Centre Hospitalier de l’Université de Montréal (CHUM) Scientific Evaluation Committee and Research Ethics Committee. All patients provided us with a written consent, available at each recruitment site. A total of 240 cases affected with SCZ were selected to constitute the case cohort. 5 cases were excluded because of their non-european ethnicity; the remainders of cases were all of European ancestry. 143 cases were recruited in Canada, 7 in France, 62 in Hungary and 23 in the United States. The clinical diagnosis were made and confirmed by an experienced psychiatrist at each site. All cases were then ascertained by a single clinical expert to assess if the individuals could be included in the study. There were 173 men and 62 women in the group of cases. The cohort was recruited to minimize the number of cases with substance abuse; only 18 cases (~7%) had a history of drug or alcohol abuse. At the time of ascertainment, the average age of the cases cohort was 35.44 +/- 10.09 and the average age of onset was 21.40 +/- 4.70. The unaffected individuals were from a European ancestry population collected in Canada. They were recruited through a population study and had no history of severe mental disorders. In total, 125 controls were men and 115 were women. 7 individuals were excluded because of non-Caucasian ancestry, leaving a total of 233 controls. The SBNO1 replication cohort was made of an additional 249 Caucasian SCZ patients (175 males and 74 females) recruited in Montreal as well as 256 individuals (120 males and 136 females) for which no psychiatric conditions were reported that were also recruited in Montreal. Inclusion and exclusion criteria for the replication cohort were identical to the discovery cohort.

### Samples preparation, quantification and digestion

DNA from blood or lymphoblastoid cell lines was extracted following standard protocols. DNA was quantified using the PICO green method and an ABI qPCR machine; quantity was adjusted to precisely 900 ng for a concentration of 20 ng/ul. Each DNA was subsequently digested, 8 samples at the time, using a mix of 8 different restriction enzymes, provided by the manufacturer (Agilent Technologies).

### Gene selection

The list of genes to be resequenced were those for which de novo mutations were identified in SCZ probands in the Girard et al. and Xu et al. exome sequencing studies[4,5] and the Awadalla et al. sequencing study[3], for a total of 62 genes. An additional set of genes encoding close interactors of the proteins encoded by genes with *de novo* mutations was added. The genes encoding these interactors were selected based on protein:protein interactions listed in the Human Protein Reference Database (HPRD) [12](Fig. A in S1 File). Node point proteins that showed two or more interactions with a protein from the core set were selected; in total 40 such genes were selected (see S1 Table for a list of all genes included on the assay).

### Design, Capture and enrichment

Haloplex probes were designed using the Haloplex design wizard tool (now replaced by the SureSelect design tool). In total, 2,041 regions were targeted for a total of 326.6 Kb. After masking of repeated regions and problematic high CG regions, a total of 316.93 Kb (97% of the target) was deemed suitable for Haloplex resequencing. The Haloplex baits covering these 2,041 regions were manufactured according to designs that were made at the Halogenomics headquarter (Uppsala, Sweden) and according to the Haloplex amplification procedures; this included the introduction of a biotin adjunct. For each individual, hybridization of the Haloplex probes was made with a pooled DNA substrate that was assembled following the eight restriction enzymes digestion step. The incubation of probes with the DNA was done overnight. Streptavidin coated magnetic beads and a magnetic stand were used to capture the biotin adjunct of the Haloplex probes; to separate beads with the hybridized products from the non-hybridized DNA material. DNA ligase was subsequently added to the hybridized DNA eluted from the beads so that each of the targeted fragments would be enclosed within a circular DNA molecules. Two steps of Haloase treatment were performed in order to carry out the “Halo” PCR. At that stage, each DNA sample became assigned to a unique DNA barcode that is based on a 96-barcode index provided by Halogenomics. Seven DNA pools constituted from unique barcodes were created.

### Sequencing

Each PCR pool was ligated to the Illumina standard adapter and loaded on a single lane from an Illumina HiSeq flow cell. Sequencing was done on an Illumina HiSeq 2000 using a paired-end mode that produced 100 bp reads. Once the sequencing was done, data from each lane was demultiplexed using the index of barcode and FASTQ files were generated for every individual.

### Alignment, enrichment assessment and variant calling

A first alignment against the whole genome revealed the capture specificity to be >99% for the first ten samples analyzed. Thus, every alignment was made using a custom reference that comprised the targeted regions and an additional 200 flanking base pairs on both 3’ and 5’ ends. Alignments were performed using BWA v.0.5.9[13] before they were saved in a BAM file format. The DepthOfCoverage module from the GATK suite was used to assess enrichment efficiency based on the defined targeted regions[14]. On average 302.47 Kb +/- 2.76 (95.43% of the effective Haloplex design) had sufficient coverage (≥20x) for high quality variant calling. Four of the 468 samples had a significantly lower coverage (with sufficient coverage for 269.16 Kb, 269.41 Kb, 283.29 Kb and 286,71 Kb); these samples were nonetheless kept for the analysis because good variant calling was still possible for ≥85% of the targeted regions. Variant calling was performed following the GATK Best Practice V2 and the GATK suite.

### False negative and false positive assessment

Using sequencing data from a previous sequencing project from the gene *SHANK3* and other genes from the S2D project (Synapse2disease; a large-scale project that aimed to identified de novo mutation in synaptic genes for schizophrenia and autism patients), we were able to evaluate the accuracy and specificity of the sequencing. For a specific genomic region, the S2D project identified 138 variants using Sanger sequencing. 135 of those 138 variants were now correctly confirmed using the Haloplex sequencing dataset, hence the false negative rate was ~2.2%. Conversely, a false positive rate was also assessed based on the variants identified in the Haloplex sequencing dataset. Out of 25 variants that were identified in Haloplex sequencing dataset, Sanger sequencing during the S2D project had not identified 5. However, after revisiting the original Sanger sequencing data of these 5 variants, it was concluded that they were all present in the sequencing traces but the calling processes missed them. Thus, we can conclude that the false positive rate is <4%.

### Kernel association testing

In order to test for difference in mutation burden, we used the Sequence Kernel Association Test (SKAT) algorithm[15]. SKAT is a statistical analysis package using a computationally efficient regression method that tests for associations between genetic variants in a region and a continuous or discontinuous trait. Variants were categorized in different sets. The first set was defined using the experimental origin of each gene (see Table 2). The second set was defined by the genes encompassing all variants. The third set was defined using all individual exon that included at least one variant. SKAT offers different parameters to give a different weight according to variation frequencies. As our main focus is rare variations, we decided to use the manual recommended settings for rare variants (B1 = 0.5, B2 = 0.5). Those parameters set full weight to rare variants (<1%) while ignoring the other variations. Statistical analyses were performed using R statistical software v.2.15.0.

### Sequenom haplotyping

Genotyping was performed in accordance with the iPLEX Gold protocol using matrix assisted laser desorption/ionisation time-of-flight (MALDI-TOF) mass spectrometry (Sequenom). Assays were designed using the latest version of AssayDesign 3 with the default parameters for the iPLEX Gold chemistry. Cleaned extension products were loaded into a mass spectrometer and peaks were identified using SpectroTYPER.

## Results

The resequencing effort was conducted on a cohort of 235 SCZ cases and 233 control individuals using the Haloplex-SureSelect method (Agilent Technologies) and a Illumina HiSeq 2000 apparatus (TsTv = 2.485, See S1 Table for a list of all coding variants). Using Sanger Sequencing, we estimated the false positive and false negative rate to be respectively ~4% and ~2.1%. Most of the variants were private or shared by only two individuals, but a number of variants (27%) were intermediate (maf between 1% and 5%) or common (maf higher than 5%) (Fig. B in S1 File). A higher number of rare variants was expected and is in accordance with recent findings from the 1K-genome project[16]. Interestingly, a total of 7 private nonsense mutations were observed in schizophrenia patients while only 2 private nonsense mutations were identified in the control group (Table 1). Two of those mutations were previously observed in dnSNP (one in cases, one in controls). If we take only the private nonsense mutations in schizophrenia patients, 6 nonsense mutations out of 8 were observed in genes found to carry DNM (75%). This is interesting, as the genes carrying DNM constitute only 61% of the number of genes on the assay. Although not statistically significant, this trend would suggest that our previous observation that there is an enrichment of noncoding private nonsense mutations in schizophrenia variants may be correct.

**Table 1:** All nonsense mutations identified in this study.

Chr	Position	ALT/REF	dbSNP	AA position	Gene	SCZ alleles	CTRL alleles
3	46729697	C/A	rs139496961	65/954	ALS2CL	3*	2*
4	123850294	C/T	0	130/894	SPATA5	1	0
6	35959504	A/G	rs145871916	292/971	SLC26A8	1	0
6	47680330	C/T	0	180/696	GPR115	1	0
6	47681891	G/T	0	304/696	GPR115	0	1
6	129649444	C/T	0	1400/3123	LAMA2	1	0
6	161160209	C/T	0	663/811	PLG	1	0
12	57579450	C/A	0	2200/4545	LRP1	1	0
16	10788449	C/T	0	94/486	TEKT5	1	0
20	33722687	C/A	rs200059987	149/542	EDEM2	0	1

*This nonsense mutation was not used to compute p-values as it is not a private mutation.

We then proceeded to test if the mutation burden was different between cases and controls. We first sought to test if any individual variant showed a positive association independently of the gene set. For this, we used a Fisher test and a Bonferroni correction adjusted to the total number of variants. No variants reached the significance threshold (data not shown). This is likely explained by the relatively small number of subjects sequenced in this study and the fact that we are looking at rare variants.

We set the SKAT parameters to account only for rare variants. The reason we decided to focus only on rare genetic variation is that a GWAS recently conducted for SCZ examined close to 10,000 individuals[17]. So it would be very surprising that our resequencing would pick associations from common or intermediate variants that would not have been detected in this recent GWAS. Also, an increase in rare variant burden is compatible with the elevation of DNM rate we previously demonstrated in SCZ.

The first mutation burden test was performed using the origins of the genes (Girard et al., Xu et al, S2D, Protein:Protein Interaction) as criteria for set definition (n = 4). We did this in order to evaluate if genes harbouring DNM have a higher mutation burden than expected (see Table 2). The three experimental dataset (Girard et al., Xu et al and S2D) yielded borderline associations (P <0.05); only the Girard et al. and the S2D datasets met the significance threshold once Bonferonni correction was applied (P<0.0125). However, when put together, the three dataset yielded a very significant association (p<0.000144). This supports the notion that we can enrich for SCZ predisposing genes by identifying DNM in affected probands. Although we could not fully estimate the population stratification, a principal component analysis using eigenvectors for the complete dataset revealed no difference between cases and controls (Fig C in S1 File). Interestingly, the dataset constituted of candidate genes found by looking at protein:protein interactions also shows a low, yet not significant p-value. Even though it is very early to draw any conclusion from this, it could mean that some genes encoding close interactors of the gene products found in DNM studies may also be involved in the disease and that the interactome approach to identify candidate genes could be a valid method.

**Table 2:** SKAT results for the dataset grouped by data source.

Set ID	P value	Number of markers per set
Girard et al.	0.0015	1073
Xu et al.	0.0260	2322
S2D Project	0.0051	491
Protein:Protein Interaction	0.0413	1686

* The significance threshold is p-value < 0.01.

**Fig 1:**
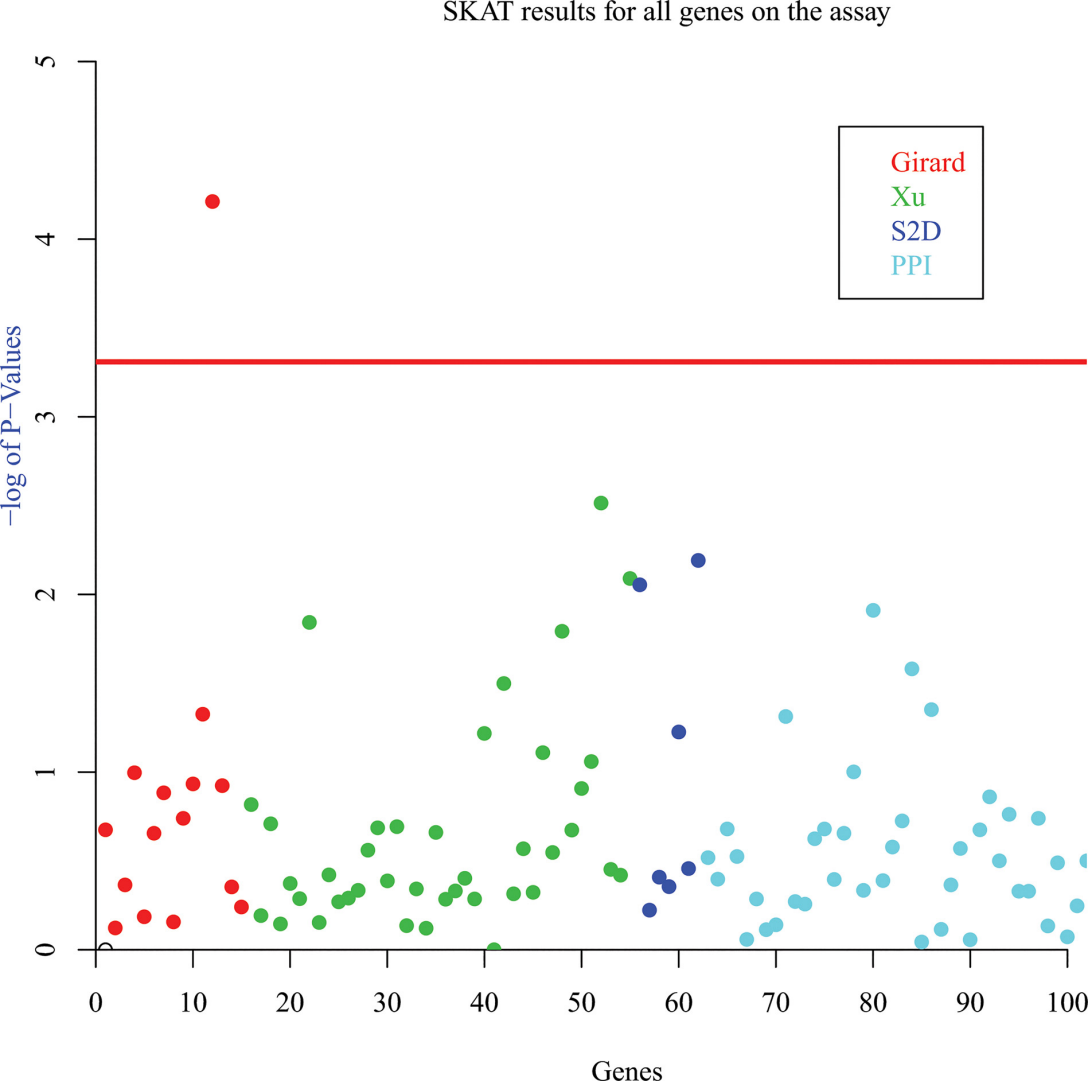
SKAT results for all genes on the assay. SKAT analysis were performed using only rare variants (< = 1%) and using genes as sets. The data sources are shown by the colors (Girard et al. = red, Xu et al. = Green, S2D project = Blue, Protein:Protein Interaction = Cyan). The significance threshold was set using a Bonferonni correction to 4.1 * 10^−4^.

**Fig 2:**
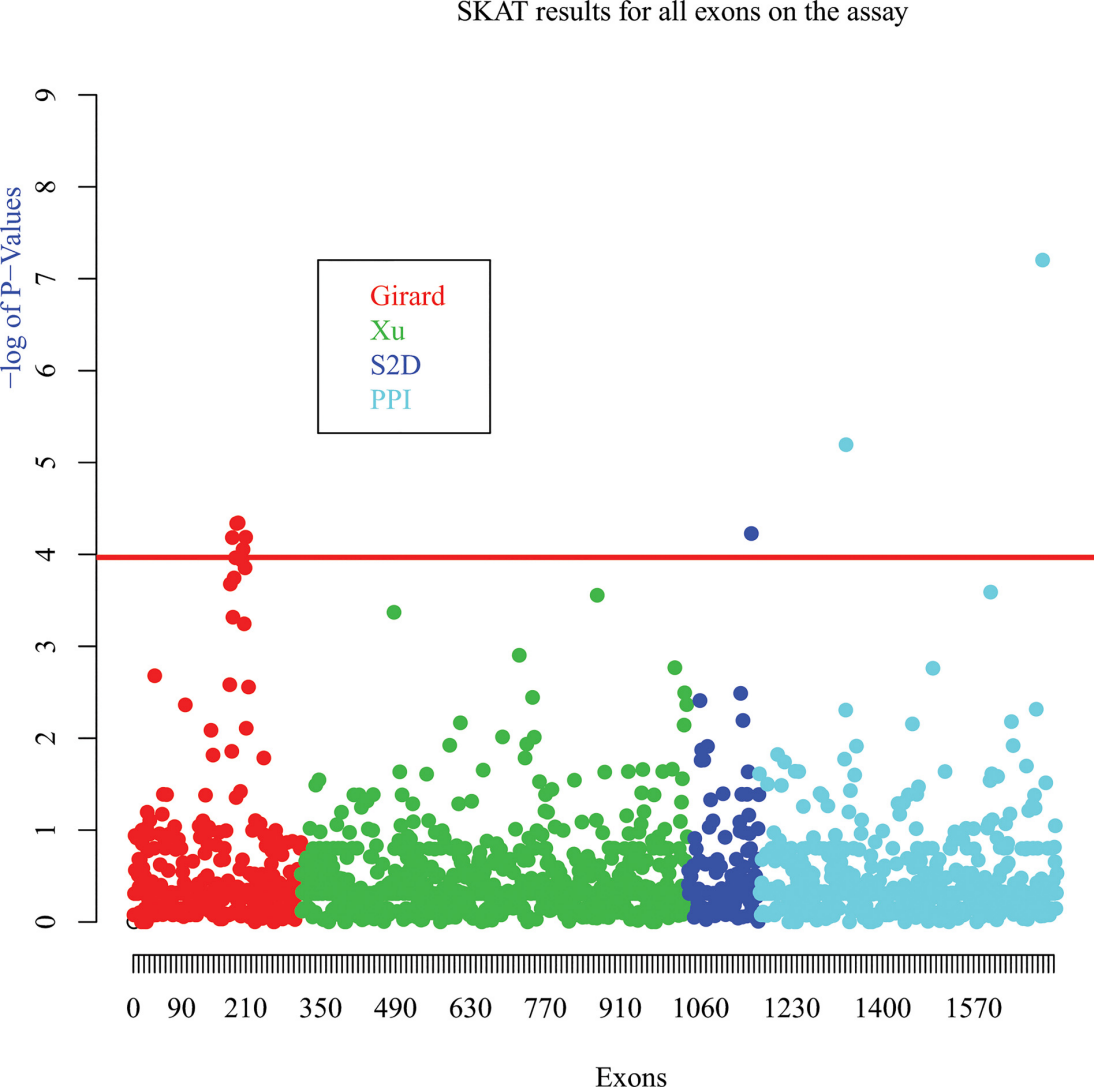
SKAT results for all exons on the assay. SKAT analysis were performed using only rare variants (< = 1%) and using exons as sets. The data sources are shown by the colors (Girard et al. = red, Xu et al. = Green, S2D project = Blue, Protein:Protein Interaction = Cyan). P-values significance threshold was set to 1.0 * 10^−4^ using simpleM method.

Next, we performed a second mutation burden test, this time treating each gene as a separate set (n = 102) and each gene was independently followed, regardless of its experimental origin. The significance threshold was set to 4.9*10^−4^ according to Bonferroni correction. Only one gene reached this significance threshold, the *strawberry notch homolog 1* (*SBNO1*) gene, which was found to carry a DNM in our earlier exome study (See Fig 1). Thus, we performed a third test, this time using single exons as separate sets. In order to correct for the number of independent SNPs, we used the same method as that used for an association study[18,19]. Using this method, the significance threshold was set to p-value < 1.08*10^−4^. This time, multiple signals met the required threshold (See Fig 2). In addition to 6 exons for *SBNO1*, we also found a different mutation burden profile in exons from the genes *EP300*, *MAPK14* and *SHANK3* (See Table 3). We reviewed the domains encompassing the three exons for the three genes. Unfortunately, nothing of interest came out of this. It is not surprising to find the *SHANK3* gene as its implication in neurodevelopmental diseases has been shown many times[20,21]. Interestingly, *EP300* encodes the p300 protein which plays a role in many tissues[22]. It has also been shown that the loss of one copy of EP300 leads to abnormal neurodevelopment[23]. Finally, the MAPK14 gene encodes for the Mitogen-activated protein kinase 14, which is heavily involved as an integration point for multiple biochemical signals involved in cellular mechanisms[24].

**Table 3:** Significant association made on a gene and exon basis.

Gene	Gene p-value	Number of markers per gene	Number of Exons	Exon p-value	Number of markers per exon
**SBNO1**	6.13*10^-5	105			
** **			30/31	6.52*10^-5	5
** **			29/31	8.80*10^-5	7
** **			28/31	4.53*10^-5	2
** **			24/31	4.62*10^-5	2
** **			13/31	0.0001	5
** **			7/31	6.52*10^-5	2
**MAPK14**	0.026	30	11/13	6.39*10^-6	2
**SHANK3**	0.0064	68	10/23	5.90*10^-5	2
**EP300**	0.0998	116	9/31	6.26*10^-8	3

*SBNO1* is the only gene that is significantly associated in the analysis looking specifically at genes. When exons are used as set, multiple exons from *SBNO1* reach the significance threshold. We looked at the individuals carrying variants in *SBNO1* and found that the signal was driven by variants shared by a subset of individuals. As SKAT is not robust when variants are in strong association, we wanted to determine if this observation was due to a shared haplotype. We genotyped all the variants that were driving the signals using a Sequenom platform on a new cohort of 249 SCZ, 256 controls ethnically matched controls. Out of the 22 variants, most individuals had none or less than 5 variants. We considered that all individuals that carried more than five variants were assumed to have the SCZ haplotype. 54 individuals with the haplotype were found in both the SCZ (21.7%) and control (21.09%) replication cohort, in agreement with the null hypothesis (p-value = 0.91). There was also no difference in the distribution of the number of variants per individual in the cases and in the controls (Fig D in S1 File). This led us to suspect that the *SBNO1* may be a false positive association found using the SKAT algorithm due to the strong LD between the variants. Thus, we performed a mutation burden study on the original cohort where all the linked variants were collapsed into one and the signal was lost. We next revisited all the associated exons for the genes SHANK3, MAPK14 and EP300 and all variants driving the signals were not shared by the same individuals, thus there is no inflation of the p due to a haplotype.

## Discussion

Many population studies in SCZ have been conducted in the last decades. From twin studies to genetic linkage and genome wide association studies, the general conclusion has always been that the genetic mechanism of SCZ is far more complex than previously estimated. Thus, we did not engage in this study expecting that we would identify determinants of a large proportion of the disease’s heritability. However, our study is different from previous studies in two ways. The first is that by relying on candidate genes for DNM, we limit the multiple testing burden to genes of potentially greater importance to the disease aetiology. There is always the risk that none of the selected genes is really involved in the disease, but many studies suggest that genes carrying DNM are good candidates. Our study is also amongst the first population study to focus only on rare variants. The contribution of rare variants has always been difficult to evaluate, but now collapsing algorithms have been developed to study the datasets generated by the recent advent of high-throughput sequencing methods. We need to keep in mind that the sample size for this current study is small. Thus, it is possible that genes with a real burden of rare variants were missed due to the fact that we were underpowered to detect more marginal association. It is also plausible that some association made in this study are artefact from population structure although we believe this unlikely, as we have shown we have no significant bias between cases and controls.

We previously identified many genes harbouring a DNM in different schizophrenia patients. However, we are still in the early days of DNM studies and it remains a challenge to demonstrate the validity of the findings. In this study, we designed a resequencing assay for genes that were reported to have DNM in SCZ (S1 Table). In addition, we also looked at the sequence of genes encoding close interactors of the genes bearing DNM. We were able to identify exons of three genes that have a mutation burden profile that is different between SCZ cases and controls. These genes include one that has been previously linked to SCZ, Autism Spectrum Disorder and Intellectual Disability (*SHANK3*) as well as two novel genes that were identified through a protein:protein interaction study (*EP300* and *MAPK14*). However, these two genes will need to be replicated in a larger cohort before we can draw a conclusion on their role in SCZ. More importantly, we were able to show that genes found in DNM studies do have a differential mutation profile in SCZ patients, either on a group scale or individual scale. The use of sets of exons for association tests with rare variants may be an analytical approach worth considering in future studies. Indeed, it has been shown that mutations leading to disease can cluster in certain specific exons of a gene[25–27]. Thus, using the full gene as set definition could lead to a loss of power from the high proportion of variants from neutral exons.

In this study, we have demonstrated that genes identified in DNM studies likely play a role in the genetic aetiology of SCZ. Our work also supports a role for rare variants in the genetic mechanism of the disease using a cost-effective method that is an interesting alternative to the sequencing of thousands of exome. Although the identified signals need replication in independent cohorts, these results call for a better integration of rare and private variants in future SCZ studies.

The data for this study is accessible through the European Nucleotide Archive (ENA) under the study PRJEB9045. The case / control / exclusion information is available in S3 Table.

## Supporting Information

S1 Table List of genes included on the resequencing assay. (DOCX)

S2 Table List of all coding variants identified in the resequencing assay. (XLSX)

S3 Table Case and control information for all individual sequenced in this study. (XLSX)

S1 File All four supplementary figures. **Fig A:** Network of genes with two or more protein:protein interactions with genes harbouring DNM, **Fig B:** Histogram of variant frequencies, **Fig C:** Principal component analysis of all samples in the cohort, **Fig D:** Additional genotyping for the SBNO1 haplotype
(DOCX)
