# Supplementary material for: Mutation Burden of Rare Variants in Schizophrenia Candidate Genes
Source: PLoS One. 2015 Jun 3;10(6):e0128988. doi: 10.1371/journal.pone.0128988 (PMC4454531; doi:10.1371/journal.pone.0128988)
Supplement: S1 Table — (DOCX) [file pone.0128988.s001.docx]

#### Supplementary Table 1 List of genes included on the resequencing assay

| **Gene ID** | **Data Source** |
| --- | --- |
| ALS2CL | Girard et al. |
| CASP4 | Girard et al. |
| CCDC137 | Girard et al. |
| CHD4 | Girard et al. |
| EIF5 | Girard et al. |
| KDM2B | Girard et al. |
| KPNA1 | Girard et al. |
| LAMA1 | Girard et al. |
| LRP1 | Girard et al. |
| NRIP1 | Girard et al. |
| PIK3CB | Girard et al. |
| SBNO1 | Girard et al. |
| SDF4 | Girard et al. |
| ZNF480 | Girard et al. |
| ZNF565 | Girard et al. |
| ACOT6 | Xu et al. |
| ADAMTS3 | Xu et al. |
| ADCY7 | Xu et al. |
| CCDC108 | Xu et al. |
| CELF2 | Xu et al. |
| COL3A1 | Xu et al. |
| DGCR2 | Xu et al. |
| DPYD | Xu et al. |
| EDEM2 | Xu et al. |
| ESAM | Xu et al. |
| FAM3D | Xu et al. |
| FASTKD5 | Xu et al. |
| GIF | Xu et al. |
| GPR115 | Xu et al. |
| GPR153 | Xu et al. |
| INPP5A | Xu et al. |
| KLF12 | Xu et al. |
| LAMA2 | Xu et al. |
| MAGEC1 | Xu et al. |
| MTOR | Xu et al. |
| NPRL2 | Xu et al. |
| OR4C46 | Xu et al. |
| PAG1 | Xu et al. |
| PITPNM1 | Xu et al. |
| PLCL2 | Xu et al. |
| PML | Xu et al. |
| RB1CC1 | Xu et al. |
| RGS12 | Xu et al. |
| SAP30BP | Xu et al. |
| SLC26A7 | Xu et al. |
| SLC26A8 | Xu et al. |
| SPATA5 | Xu et al. |
| TEKT5 | Xu et al. |
| THBS1 | Xu et al. |
| TRAK1 | Xu et al. |
| TRRAP | Xu et al. |
| UGT1A3 | Xu et al. |
| VPS35 | Xu et al. |
| WDR11 | Xu et al. |
| ZNF530 | Xu et al. |
| ATP2B4 | S2D project |
| BSN | S2D project |
| GRIN2B | S2D project |
| KIF17 | S2D project |
| MAP2K1 | S2D project |
| NRXN1 | S2D project |
| SHANK3 | S2D project |
| APBA1 | Protein:Protein Interaction |
| APBA2 | Protein:Protein Interaction |
| APP | Protein:Protein Interaction |
| C3 | Protein:Protein Interaction |
| CALR | Protein:Protein Interaction |
| CAPN1 | Protein:Protein Interaction |
| CASK | Protein:Protein Interaction |
| CCDC85B | Protein:Protein Interaction |
| COL7A1 | Protein:Protein Interaction |
| CTBP1 | Protein:Protein Interaction |
| CTSG | Protein:Protein Interaction |
| DLG1 | Protein:Protein Interaction |
| DLG2 | Protein:Protein Interaction |
| DLG3 | Protein:Protein Interaction |
| DLG4 | Protein:Protein Interaction |
| EP300 | Protein:Protein Interaction |
| ESR1 | Protein:Protein Interaction |
| FYN | Protein:Protein Interaction |
| HDAC1 | Protein:Protein Interaction |
| HDAC3 | Protein:Protein Interaction |
| JUN | Protein:Protein Interaction |
| MAPK14 | Protein:Protein Interaction |
| MYC | Protein:Protein Interaction |
| PDGFB | Protein:Protein Interaction |
| PLAT | Protein:Protein Interaction |
| PLAUR | Protein:Protein Interaction |
| PLCB1 | Protein:Protein Interaction |
| PLG | Protein:Protein Interaction |
| PRKCD | Protein:Protein Interaction |
| PTPN11 | Protein:Protein Interaction |
| RARA | Protein:Protein Interaction |
| SHC1 | Protein:Protein Interaction |
| SIN3A | Protein:Protein Interaction |
| SKIL | Protein:Protein Interaction |
| SPARC | Protein:Protein Interaction |
| SRC | Protein:Protein Interaction |
| STAT3 | Protein:Protein Interaction |
| TAF9 | Protein:Protein Interaction |
| TRIM27 | Protein:Protein Interaction |
| WNT3A | Protein:Protein Interaction |
