# Supplementary material for: Mutation Burden of Rare Variants in Schizophrenia Candidate Genes
Source: PLoS One. 2015 Jun 3;10(6):e0128988. doi: 10.1371/journal.pone.0128988 (PMC4454531; doi:10.1371/journal.pone.0128988)

**Supplementary Figure A: Network of genes with two or more protein:protein interactions with genes harbouring DNM** : This figure shows the interaction between some DNM harbouring genes (yellow) and close interactors (cyan). The list of DNM harbouring genes was those identified in Girard et al., Xu et al. and the 7 genes identified during the synapse2disease projet (S2D) (Girard, 2011, Xu 2011, Awadalla et al 2010). The protein:protein interactions were those found in the Human Protein Reference Database (HPRD). All genes that showed two or more interactions with DNM gene set were kept. In total, 40 interacting genes met this criterion.

**Supplementary Figure B: Histogram of variant frequencies** : For each variant identified in the Haloplex dataset, we established a minor allele frequency. We then established a histogram of those frequencies. In the pie chart, the definition goes as following for variants : common (>5%), intermediate (1-5%), rare (0.5-1%) and very rare (<0.5%)

**Supplementary Figure C: Principal component analysis of all samples in the cohort** : Using all identified variants, we did a principal component analysis using SNPRelate R package. Y axis represents the first component (eigenvector) and X axis represents the second eigenvector.

**Supplementary Figure D: Additional genotyping for the SBNO1 haplotype** Sequenom genotyping was used to test for the SBNO1 haplotype in an additional cohort of SCZ and controls. Panel A shows the exact distribution of the number of variants per individuals per cohorts and Panel B shows a summary of the same distributions.

Supplementary figure A


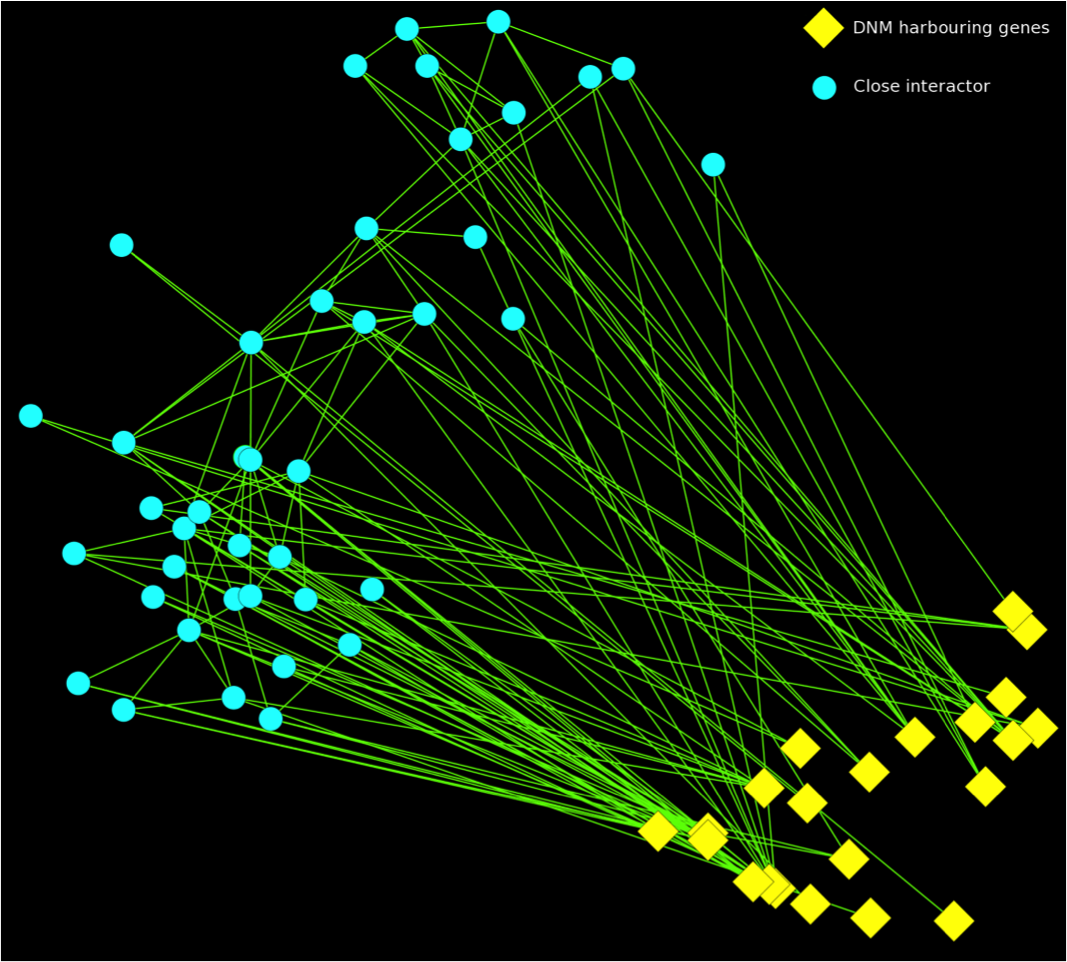


Supplementary Figure B


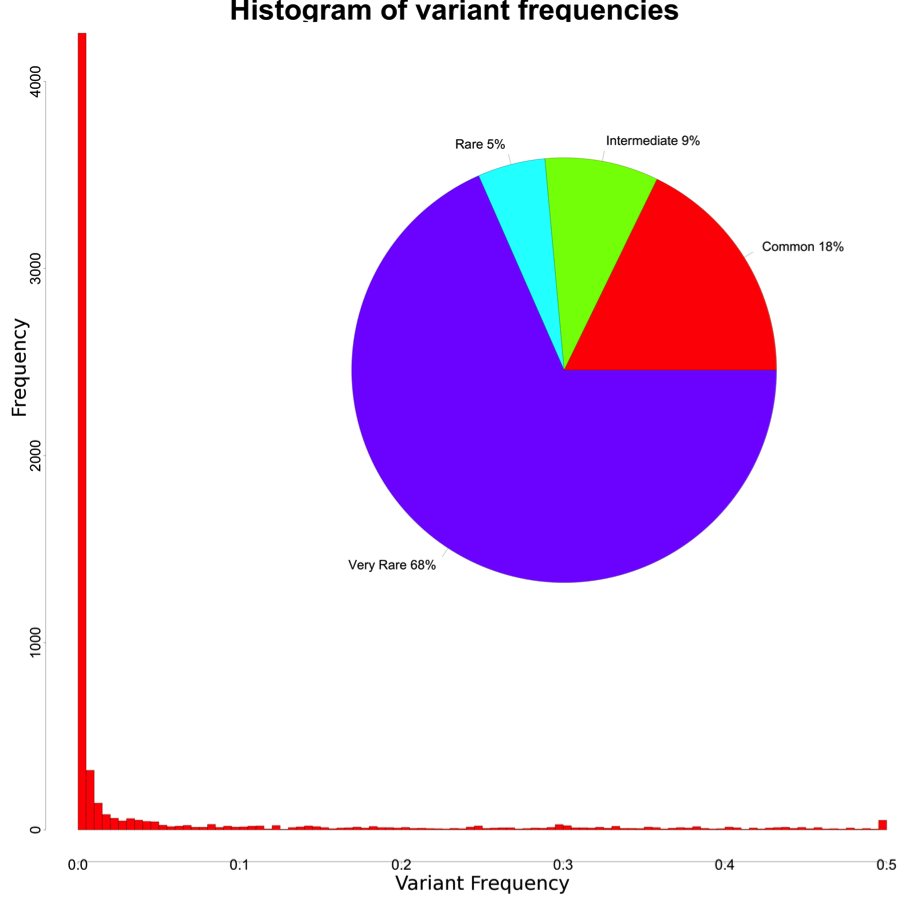


Supplementary Figure C


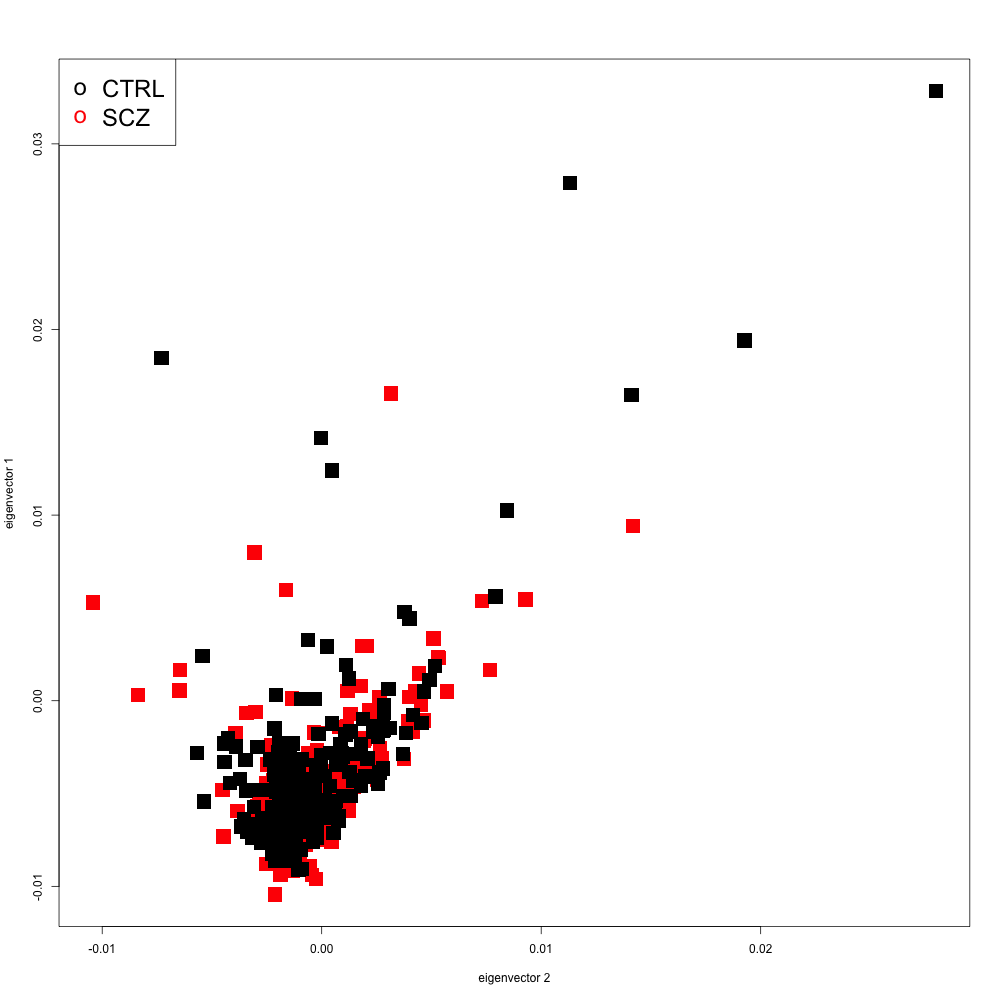


Supplementary Figure D


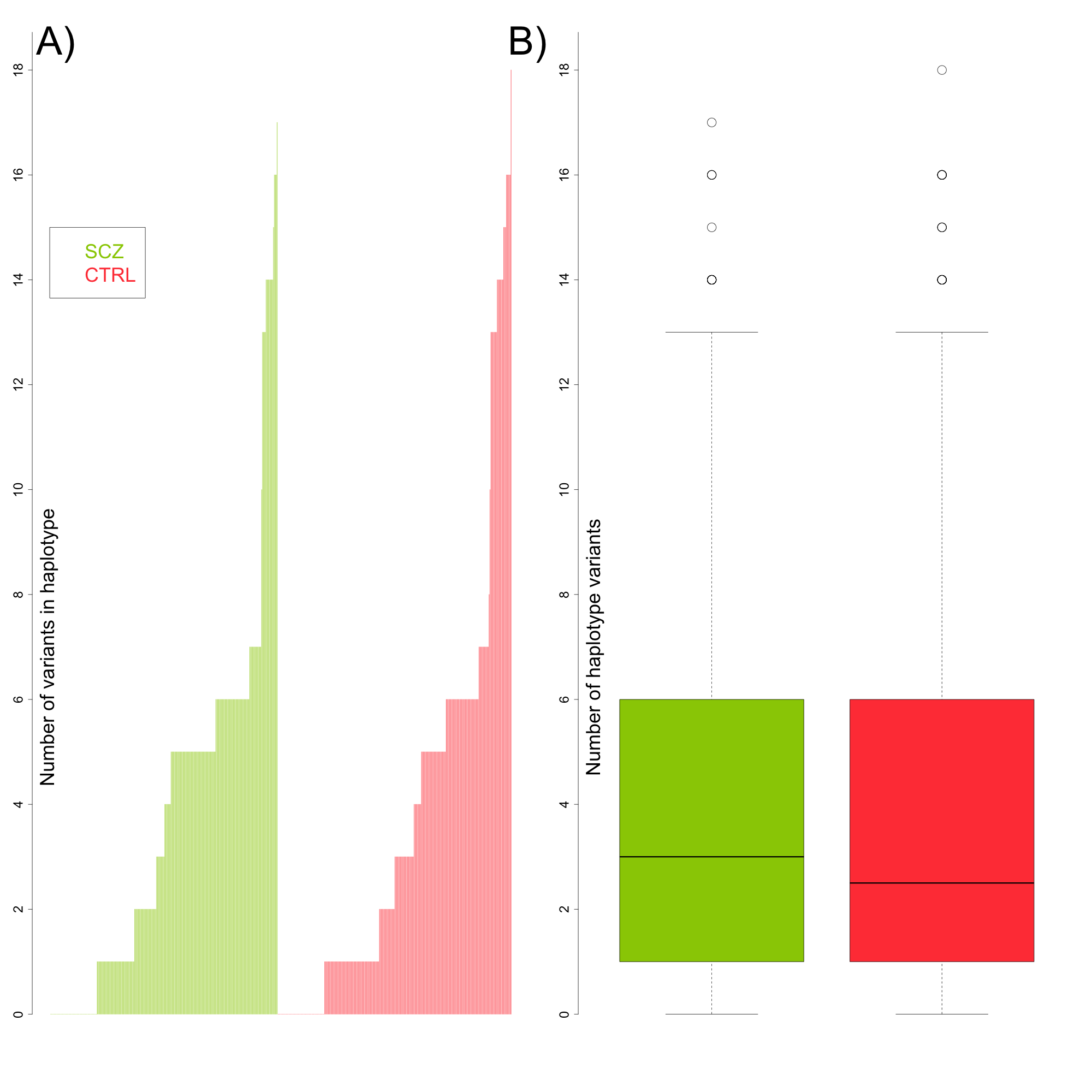

Supplement: S1 File — Fig A: Network of genes with two or more protein:protein interactions with genes harbouring DNM, Fig B: Histogram of variant frequencies, Fig C: Principal component analysis of all samples in the cohort, Fig D: Additional genotyping for the SBNO1 haplotype (DOCX) [file pone.0128988.s004.docx]
